# Exploring the relationship between atorvastatin and rosuvastatin use and respiratory, thoracic, and mediastinal disorders: A retrospective study

**DOI:** 10.1097/MD.0000000000044984

**Published:** 2025-10-10

**Authors:** Tian Tan, Yunhong Lei, Cuixian Li, Yi Zhang, Jinglin Song, Rong Zhang

**Affiliations:** a Xiangyang Central Hospital, Affiliated Hospital of Hubei University of Arts and Science, Hubei Province, China; b Yichang Hubo Medical Research Institute, Hubei Province, China; c The Department of Dermatology, The First Affiliated Hospital of Dali University, Yunnan Province, China; d Department of Nursing, Pingdingshan University, Henan Province, China; e Philippine Women’s University, Manila, Philippines.

**Keywords:** atorvastatin, FAERS, pharmacovigilance, respiratory, rosuvastatin, signal detection, thoracic and mediastinal disorders

## Abstract

This study aimed to comprehensively evaluate the risk of respiratory, thoracic and mediastinal disorders associated with atorvastatin and rosuvastatin use. We conducted a retrospective pharmacovigilance study using the FDA Adverse Event Reporting System (FAERS) database from Q1 2014 to Q1 2023. Disproportionality analysis was performed to quantify the risk of respiratory, thoracic and mediastinal disorders, using the reporting odds ratio (ROR), confidence interval (CI), information component (IC), and its lower 95% credibility interval (IC_025_). We also assessed the time to onset of these adverse events. We identified a total of 15,676 reports of respiratory, thoracic and mediastinal disorders linked to statins were identified. Atorvastatin (ROR = 2.05, 95% CI: 2.02–2.08, IC = 0.96, IC_025_ = 0.93) and rosuvastatin (ROR = 1.90, 95% CI: 1.87–1.93, IC = 0.86, IC_025_ = 0.82) both demonstrated significant associations with these adverse events. At the preferred term (PT) level, 63 positive signals were detected, with bronchial irritation (ROR = 43.31, 95% CI: 25.61–73.24; IC = 5.24, IC_025_ = 4.49) and prolonged expiration (ROR = 27.44, 95% CI: 16.92–44.50; IC = 4.65, IC_025_ = 3.90) showing the strongest signals. Middle-aged and elderly women (18–74 years) and elderly men (≥75 years) appeared to be at higher risk of developing respiratory, thoracic, and mediastinal disorders following statin use. With respect to the time to onset of these disorders, atorvastatin was associated with a median onset of 7 days, whereas rosuvastatin showed a longer median onset time of 14.5 days. This study suggests a significant association between atorvastatin and rosuvastatin and respiratory, thoracic, and mediastinal disorders. Overall, atorvastatin was associated with a higher risk; however, rosuvastatin exhibited stronger disproportionate signals in certain specific respiratory events, such as catarrh and nasal edema. Further studies are warranted to validate these findings and to elucidate the underlying mechanisms.

## 1. Introduction

Elevated low-density lipoprotein cholesterol is a well-established risk factor for the development and progression of cardiovascular disease.^[1]^ The enzyme hydroxy-3-methylglutaryl coenzyme A reductase serves as the primary rate-limiting step in de novo cholesterol synthesis, significantly influencing systemic cholesterol levels.^[2]^ Statins, a class of hydroxy-3-methylglutaryl coenzyme A reductase inhibitors, are widely used to manage hypercholesterolemia due to their robust clinical efficacy and well-characterized safety profiles.^[3,4]^ As a cornerstone of lipid-lowering therapy, statins have been among the most frequently prescribed medications globally for decades.^[5]^

Despite their efficacy in cholesterol management, concerns persist regarding statin-associated adverse events, particularly at higher doses (e.g., atorvastatin ≥ 80 mg/day, rosuvastatin ≥ 80 mg/day).^[6]^ These adverse events include elevated aminotransferase levels, cholestasis, and muscle-related toxicities such as myalgia or, in severe cases, rhabdomyolysis, which can significantly affect patient adherence.^[7]^ In recent years, emerging case reports have highlighted potential respiratory and pulmonary adverse events linked to statin use.^[8]^ Notably, Fernández et al identified interstitial lung disease as a possible novel adverse event associated with most statins.^[9]^ Other reported respiratory complications include dyspnea, cough, and interstitial lung disease, though these events are rare. Given their potential impact on patient health and treatment compliance, these adverse effects warrant thorough investigation.^[2,10]^

The FDA Adverse Event Reporting System (FAERS) is a multicentric, large-scale, dynamically updated, and publicly accessible post-marketing drug safety surveillance database in the United States.^[11,12]^ This study leverages FAERS data to evaluate the association between atorvastatin and rosuvastatin and respiratory, thoracic and mediastinal disorders, characterize at-risk patient populations, and analyze temporal patterns of these events using established pharmacovigilance methods for adverse event signal detection.

## 2. Method

### 2.1. Data sources

This retrospective, observational pharmacovigilance study evaluated the association between statins and respiratory, thoracic and mediastinal disorders using real-world data from FAERS. The analysis included all FAERS data from the first quarter of 2014 to the first quarter of 2023. The FAERS database comprises 7 data tables: DEMO: Provides patient demographic and administrative information; DRUG: Details drug-related information; REAC: Documents adverse event information; OUTC: Records patient outcome data; RPSR: Identifies the source of the report; THER: Specifies treatment start and end dates for reported drugs; INDI: Lists indications for drug use. During data preprocessing, duplicate reports were removed following FDA-recommended methodology. Reports were sorted by PRIMARYID, CASEID, and FDA_DT fields from the DEMO table. For reports with identical CASEID, the record with the most recent FDA_DT was retained. If both CASEID and FDA_DT were identical, the report with the highest PRIMARYID was kept. Since the FAERS database is publicly accessible and contains anonymized and de-identified patient records, this study does not require informed consent or ethical approval. The raw FAERS data were cleaned, mapped, deduplicated, and normalized using R software (version 3.6.1).

### 2.2. Definition of suspected drug

Statins were identified using the World Health Organization anatomical therapeutic chemical classification system (code C10AA). This study focused on 2 widely used statins: atorvastatin (C10AA05) and rosuvastatin (C10AA07). In the FAERS DRUG table, drugs are classified into 4 roles: primary suspect drug (PS), secondary suspect drug (SS), concomitant (C), and interacting (I). To minimize confounding in causality assessment, only reports designating statins as the primary suspect (PS) drug were included.

### 2.3. Definition of adverse events

Adverse events in the FAERS database are coded using preferred terms (PTs) from the Medical Dictionary for Regulatory Activities (MedDRA, version 24.1), which are grouped into system organ classes. This study targeted adverse events classified under the system organ class (SOC) “Respiratory, Thoracic, and mediastinal disorders” (MedDRA code: 10038738).

### 2.4. Signal detection

This study employed a case/non-case design, where reports of respiratory, thoracic and mediastinal disorders were designated as “cases,” and all other reports as “non-cases.” Two disproportionality methods were used to detect potential signals of association between statins and adverse events: the reporting odds ratio (ROR) and the Bayesian Confidence Propagation Neural Network. ROR: A signal was considered detected if the lower limit of the 95% confidence interval (CI) exceeded 1 and at least 10 cases were reported. Higher ROR values indicate stronger signals.^[13]^ Information Component (IC) of Bayesian Confidence Propagation Neural Network: A signal was detected if the lower limit of the 95% CI was >0.

An adverse event was deemed a signal only if both ROR and IC methods indicated significance. The algorithms for ROR and IC calculations are provided in Table 1.^[14]^ Additionally, 2 subgroup analyses were conducted using ROR to evaluate the specificity of drug-adverse event associations across age and sex groups. Cases with missing sex or age information were categorized as “unknown.” These reports were excluded from subgroup and cross-stratification analyses, and only cases with complete demographic data were considered in the final analysis.

**Table 1:** The specific formulas for the 2 algorithms are as follows.

Algorithms	Equation	Criteria
ROR	ROR = ad/bc	lower limit of 95% CI > 1, N ≥ 10
	95% CI = e^ln(ROR)±1.96 (1/a+1/b+1/c+1/d)^0.5^	
BCPNN	IC = log_2_^a(a+b+c+d)/[(a+c)(a+b)]^	IC_025_ > 0
	95% CI = E(IC) ± 2 [V(IC)]^0.5^	

“a” represents number of reports containing both the target drug and the target adverse drug reaction; “b” represents the number of reports containing other adverse drug reactions of the target drug; “c” represents the number of reports containing the target adverse drug reaction of other drugs; “d” represents the number of reports containing other drugs and other adverse drug reactions.

BCPNN = Bayesian Confidence Propagation Neural Network , CI = confidence interval, IC = information component, ROR = reporting odds ratio.

## 3. Results

### 3.1. Data overview

Between Q1 2014 and Q1 2023, a total of 21,114,357 adverse event reports were retrieved from the FAERS database. After removing duplicate entries, 15,676 reports related to respiratory, thoracic and mediastinal disorders were identified, of which 8211 were associated with atorvastatin and 7465 with rosuvastatin.

### 3.2. Clinical characteristics of cases

The clinical characteristics of patients who experienced respiratory, thoracic and mediastinal disorders while using statins are summarized in Table 2. A majority of the reported cases involved female patients, and 59.17% were aged between 18 and 75 years. The median age across all patients was approximately 66 years, with median ages of 65 years for atorvastatin users and 69 years for rosuvastatin users. Most reports originated from the United States, while a smaller proportion were from Canada. The Canadian reports were most likely submitted by pharmaceutical manufacturers, with adverse events occurring in Canada but reported in accordance with pharmacovigilance obligations under U.S. regulations.

**Table 2:** Demographic information on respiratory, thoracic and mediastinal disorders with statins.

Characteristic	Cases, N (%)				
	Atorvastatin		Rosuvastatin		Total
Total cases	8211 (52.38%)		7465 (47.62%)		15,676
Gender					
Female	4540 (55.29%)		3567 (47.78%)		8107 (51.72%)
Male	2943 (35.84%)		3285 (44.01%)		6228 (39.73%)
Unknown	734 (8.94%)		613 (8.21%)		1347 (8.59%)
Age					
<18	50 (0.61%)		91 (1.22%)		141 (0.90%)
18–75	5726 (69.74%)		3549 (47.54%)		9275 (59.17%)
>75	1254 (15.27%)		2031 (27.21%)		3285 (20.96%)
Median (IQR)	65 (61–74)		69 (60–78)		66 (60–76)
Reported countries (top 3)	Canada	4239 (51.63%)	Canada	4855 (65.04%)	9094 (58.01%)
	United States	852 (10.38%)	United States	723 (9.69%)	1575 (10.05%)
	Great Britain	504 (6.14%)	Germany	651 (8.72%)	1155 (7.37%)
Outcomes	Hospitalization	3582 (43.62%)	Other serious/important medical event	4403 (58.98%)	7985 (50.94%)
	Other serious/important medical event	3523 (42.91%)	Hospitalization	2352 (31.51%)	5875 (37.48%)
	Death	438 (5.33%)	Disability	202 (2.71%)	640 (4.08%)
	Life-threatening	218 (2.65%)	Congenital anomaly	110 (1.47%)	328 (2.09%)
	Disability	159 (1.94%)	Life-threatening	91 (1.22%)	250 (1.59%)
Reported year					
2016	233 (2.84%)		179 (2.40%)		412 (2.63%)
2017	263 (3.20%)		190 (2.55%)		453 (2.89%)
2018	469 (5.71%)		268 (3.59%)		737 (4.70%)
2019	813 (9.90%)		411 (5.51%)		1224 (7.81%)
2020	945 (11.51%)		565 (7.57%)		1510 (9.63%)
2021	2103 (25.61%)		1634 (21.89%)		3737 (23.84%)
2022	3218 (39.19%)		3151 (42.21%)		6369 (40.63%)
2023	686 (8.35%)		1067 (14.29%)		1753 (11.18%)

Regarding clinical outcomes, hospitalization was the most commonly reported outcome among atorvastatin-associated cases, followed by other serious outcomes and death. In contrast, for rosuvastatin, the most frequent outcome was other serious serious outcomes, followed by hospitalization and disability.

### 3.3. Disproportionality analysis of respiratory, thoracic and mediastinal disorders

We quantified the frequency and types of adverse events within the respiratory, thoracic and mediastinal disorders (SOC code in MedDRA: 10038738) for each statin. Both atorvastatin and rosuvastatin demonstrated significant signals: Atorvastatin: ROR = 2.05 (95% CI: 2.02–2.80), IC = 0.96, IC_025_ = 0.93; Rosuvastatin: ROR = 1.90 (95% CI: 1.87–1.93), IC = 0.86, IC_025_ = 0.82.

The 5 most frequently reported PTs for atorvastatin-related respiratory, thoracic and mediastinal disorders were: Dyspnoea (1741 cases), Cough (772 cases), Interstitial lung disease (366 cases), Exertional dyspnea (252 cases), Oropharyngeal pain (237 cases) (Table 3). For rosuvastatin, the top 5 PTs were: Dyspnoea (1231 cases), Asthma (768 cases), Cough (648 cases), Wheeze (460 cases), Exertional dyspnea (225 cases) (Table 4).

**Table 3:** The number of reports on respiratory, thoracic, and mediastinal disorders associated with atorvastatin at the preferred term (PT) level in the FAERS database.

Preferred terms (PTs)	Case numbers
Dyspnoea	1741 (21.20%)
Cough	772 (9.40%)
Interstitial lung disease	366 (4.46%)
Dyspnoea exertional	252 (3.07%)
Oropharyngeal pain	237 (2.89%)

FAERS = FDA Adverse Event Reporting System.

**Table 4:** The number of reports on respiratory, thoracic, and mediastinal disorders associated with rosuvastatin at the preferred term (PT) level in the FAERS database.

Preferred terms (PTs)	Case numbers
Dyspnoea	1231 (16.49%)
Asthma	768 (10.29%)
Cough	648 (8.68%)
Wheezing	460 (6.16%)
Dyspnoea exertional	225 (3.01%)

FAERS = FDA Adverse Event Reporting System.

Further disproportionality analysis was conducted at the PT level using the entire FAERS database as the comparator. Among atorvastatin-related reports, the top 3 PTs with the strongest signals were: Bronchial irritation: ROR = 43.31 (95% CI: 25.61–73.24); IC = 5.24, IC_025_ = 4.49; Prolonged expiration: ROR = 27.44 (95% CI: 16.92–44.50); IC = 4.65, IC_025_ = 3.96; Pulmonary vascular disorder: ROR = 27.39 (95% CI: 18.32–40.94); IC = 4.65, IC_025_ = 4.07 (Table S1, Supplemental Digital Content, https://links.lww.com/MD/Q285). For rosuvastatin, the strongest PT signals were: Catarrh: ROR = 56.74 (95% CI: 44.68–72.04); IC = 5.67, IC_025_ = 5.33; Nasal edema: ROR = 35.90 (95% CI: 28.26–45.59); IC = 4.72, IC_025_ = 4.40; Chronic respiratory disease: ROR = 31.03 (95% CI: 18.42–52.27); IC = 4.87, IC_025_ = 4.13 (Table S2, Supplemental Digital Content, https://links.lww.com/MD/Q285). Although atorvastatin exhibited a higher risk profile at the SOC level, rosuvastatin demonstrated stronger disproportionate signals in certain specific PTs (e.g., catarrh and nasal edema), highlighting potential differences in the adverse event spectra between the 2 statins.

### 3.4. Specific risk differences

Subgroup analysis by sex and age revealed notable disparities. Females exhibited a higher risk of respiratory, thoracic and mediastinal disorders for both statins: Atorvastatin: ROR = 1.29 (95% CI: 1.18–1.40); Rosuvastatin: ROR = 4.02 (95% CI: 3.39–4.76). In addition, patients aged over 75 years demonstrated increased susceptibility, particularly for atorvastatin (ROR = 1.90, 95% CI: 1.38–2.61). These findings underscore the need for clinicians to account for sex- and age-specific differences when prescribing statins, as elderly patients and females appear more prone to develop respiratory, thoracic and mediastinal disorders (Table 5).

**Table 5:** Sex and age differences in atorvastatin and rosuvastatin-related adverse events.

SOC	Atorvastatin (ROR, 95% CI)				Rosuvastatin (ROR, 95% CI)			
	Female	Male	18–74	75	Female	Male	18–74	75
Respiratory, thoracic and mediastinal disorders	1.29 (1.18–1.4)	1.22 (1.11–1.34)	1.16 (1.07–1.26)	1.9 (1.38–2.61)	4.02 (3.39–4.76)	1.71 (1.6–1.83)	1.48 (1.4–1.57)	1.62(1.49–1.76)

ROR = reporting odds ratio, SOC = system organ class.

To further investigate the impact of sex and age on the association between drug use and adverse events, we conducted a cross-stratification analysis, with results presented in Table 6. Using middle-aged and elderly men (18–74 years) as the reference group, both middle-aged and elderly women (18–74 years) and elderly women (≥75 years) showed stronger associations between atorvastatin or rosuvastatin use and respiratory, thoracic, and mediastinal disorders, particularly for rosuvastatin. However, when compared with women in both age groups (18–74 years and ≥75 years), elderly men (≥75 years) exhibited stronger associations with these disorders following atorvastatin or rosuvastatin use.

**Table 6:** The cross-stratification base on sex and age of atorvastatin and rosuvastatin.

Drug	Patients	Reference	ROR (95% CI)
Atorvastatin	Middle-aged and elderly female	Middle-aged and elderly men	1.06 (1.02–1.11)
	Elderly female		1.23 (1.16–1.30)
	Middle-aged and elderly female	Elderly men	0.80 (0.75–0.86)
	Elderly female		0.70 (0.66–0.74)
Rosuvastatin	Middle-aged and elderly female	Middle-aged and elderly men	1.60 (1.51–1.69)
	Elderly female		2.14 (2.02–2.26)
	Middle-aged and elderly female	Elderly men	0.79 (0.75–0.82)
	Elderly female		0.18 (0.17–0.19)

Middle-aged and elderly female: 18–74 yr-old female; Elderly female: ≥75 yr-old female; Middle-aged and elderly men: 18–74 yr-old male; Elderly men: ≥75 yr-old male.

CI = confidence interval, ROR = reporting odds ratio.

### 3.5. Time to onset of adverse events

**Figure 1.**
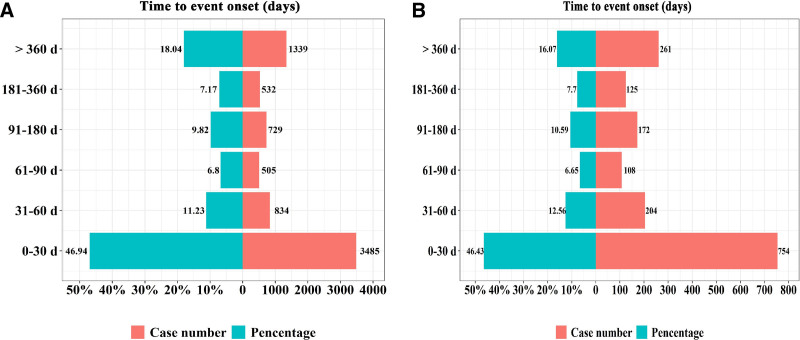
The time to onset of respiratory, thoracic and mediastinal disorders.

The time to onset of respiratory, thoracic and mediastinal disorders is illustrated in Figure 1. Based on available data from START_DT and EVENT_DT, the median time to onset for atorvastatin was 7 days (IQR: 0.5–51.5 days), suggesting a relatively early occurrence (Fig. 1A). In contrast, the median onset time for rosuvastatin was longer at 14.5 days (IQR: 3.25–723.5 days), indicating a broader temporal range for adverse event development (Fig. 1B).

## 4. Discussion

Statins are the mainstay treatment for hypercholesterolemia and are widely used worldwide. While they are clinically effective in reducing cardiovascular risk, an increasing number of studies and case reports in recent years have raised concerns about their potential to induce adverse events, particularly involving the respiratory and mediastinal system (e.g., dyspnea, cough, interstitial lung disease). This study investigated the association between statins and respiratory, thoracic and mediastinal disorders using the FAERS database, aiming to provide a more comprehensive evaluation of their safety profile.

We identified a total of 15,676 reports of respiratory, thoracic and mediastinal disorders associated with statins. Among them, 8211 cases were linked to atorvastatin and 7465 to rosuvastatin. Disproportionality analysis revealed significant associations for both drugs. The ROR for atorvastatin was 2.05 (95% CI: 2.02–2.08), and for rosuvastatin, it was 1.90 (95% CI: 1.87–1.93), suggesting a robust signal for these adverse events. The high volume of reports associated with atorvastatin, coupled with elevated signal values, indicates a potentially stronger association. The most frequently reported events for rosuvastatin were dyspnea (1231 cases) and asthma (768 cases), reinforcing the relevance of respiratory and mediastinal system involvement. These findings suggest that respiratory, thoracic and mediastinal disorders are more commonly reported with statin use than with other drugs or background levels. However, while disproportionality signals highlight possible risks, they do not confirm causality, which must be further validated by clinical trials or in-depth case reviews.

Sex-and age-specific analyses revealed additional insights. Female patients demonstrated higher signal values than males for both atorvastatin (female: ROR = 1.29, 95% CI: 1.18–1.40; male: ROR = 1.22, 95% CI: 1.11–1.34) and rosuvastatin (female: ROR = 4.02, 95% CI: 3.39–4.76; male: ROR = 1.71, 95% CI: 1.60–1.83). This sex-based difference may be attributed to physiological factors such as hormonal differences and immune response variability,^[15,16]^ which may increase susceptibility to respiratory complications in women following statin use. Regarding age, elderly patients (>75 years) were at a notably higher risk of developing respiratory, thoracic and mediastinal disorders, particularly with atorvastatin (ROR = 1.90, 95% CI: 1.38–2.61). This increased risk could be due to age-related comorbidities, such as chronic obstructive pulmonary disease or other preexisting respiratory conditions,^[17]^ which may predispose this population to adverse reactions. Cross-stratification by age and sex further demonstrated that among the very elderly population (≥75 years), men had a higher risk of developing respiratory, thoracic, and mediastinal disorders, whereas among middle-aged and elderly individuals (18–74 years), women were at higher risk, particularly with rosuvastatin use. These findings suggest that sex-related differences in adverse event risk may vary with age. Middle-aged and elderly women receiving rosuvastatin may require closer monitoring for adverse events, while very elderly men appear to be at greater risk with both drugs. Hence, clinicians should consider individual patient characteristics when prescribing statins, including sex, age, and comorbidities. In cases of severe adverse events, personalized treatment strategies and enhanced monitoring, including potential dose adjustments or alternative therapies, may be warranted.

This study also examined the time-to-onset of respiratory, thoracic and mediastinal disorders. The median time to onset was 7 days (IQR: 0.5–51.5) for atorvastatin and 14.5 days (IQR: 3.25–723.5) for rosuvastatin. This suggests that atorvastatin-associated events may manifest earlier than those of rosuvastatin. The discrepancy may be related to pharmacokinetic differences; atorvastatin has a shorter half-life compared to rosuvastatin, potentially contributing to earlier symptom onset, while rosuvastatin’s longer duration of action may delay the manifestation of adverse effects.^[3]^ These findings have important clinical implications. They highlight the need for close monitoring of respiratory and mediastinal symptoms, particularly within the first few weeks of treatment, to allow for timely intervention and mitigation of health risks.

This study has several limitations that should be acknowledged. First, the FAERS database lacks detailed clinical information, such as smoking history, occupational exposure, and concomitant medications, all of which are highly relevant to outcomes and may serve as important confounders. However, disproportionality analyses cannot adjust for such potential confounding factors, which may limit the accuracy of the ROR estimates. Second, underreporting bias is an inherent limitation of spontaneous reporting systems, as only a small fraction of adverse events are reported, and reporting may vary across populations, time periods, or drug classes. Third, missing demographic information (e.g., sex was unknown in 8.59% of cases) could introduce bias in subgroup analyses if such data are not missing at random. In addition, due to substantial missing data on drug dosage and treatment duration, we were unable to perform cross-stratification based on these parameters, which is a limitation. Finally, as a retrospective pharmacovigilance analysis, this study can only suggest associations rather than establish causality. Therefore, prospective clinical studies and large-scale cohort investigations are warranted to further validate these findings. Taken together, these limitations indicate that our results should be interpreted as hypothesis-generating, underscoring the need for further pharmacoepidemiological and mechanistic research to confirm these associations.

In conclusion, this study identified significant associations between atorvastatin and rosuvastatin use and respiratory, thoracic, and mediastinal disorders. The overall risk was higher with atorvastatin; however, rosuvastatin exhibited stronger disproportionality signals for certain specific respiratory events, such as catarrh and nasal edema. The risks appeared to be more pronounced in middle-aged and elderly women as well as in older men. The relatively short median time to onset, particularly for atorvastatin, highlights the necessity of early surveillance following the initiation of statin therapy. These findings underscore the importance of individualized risk assessment and careful monitoring in statin prescribing. Future investigations should focus on elucidating the underlying mechanisms and establishing causal relationships through rigorous clinical study designs.

## Author contributions

**Conceptualization:** Tian Tan.

**Data curation:** Cuixian Li, Yi Zhang, Rong Zhang.

**Formal analysis:** Yunhong Lei, Cuixian Li, Rong Zhang.

**Project administration:** Yunhong Lei.

**Visualization:** Yi Zhang, Jinglin Song.

**Writing – original draft:** Tian Tan.

**Writing – review & editing:** Yunhong Lei, Yi Zhang, Jinglin Song, Rong Zhang.

## Supplementary Material


