# Supplementary material for: Exploring the relationship between atorvastatin and rosuvastatin use and respiratory, thoracic, and mediastinal disorders: A retrospective study
Source: Medicine (Baltimore). 2025 Oct 10;104(41):e44984. doi: 10.1097/MD.0000000000044984 (PMC12517866; doi:10.1097/MD.0000000000044984)
Supplement: Supplementary file 1 [file medi-104-e44984-s001.docx]

Supplement Table 1. Signal strength of reports of atorvastatin at the preferred term (PT) level in FAERS database.

| Preferred terms (PTs) | Case Numbers | ROR (95%Cl) | IC (IC_025_) |
| --- | --- | --- | --- |
| Dyspnoea | 1741 (21.20%) | 1.31 (1.25 - 1.37) | 0.38 (0.31) |
| Cough | 772 (9.40%) | 1.11 (1.03 - 1.19) | 0.15 (0.05) |
| Interstitial Lung Disease | 366 (4.46%) | 3.32 (3 - 3.68) | 1.72 (1.57) |
| Dyspnoea Exertional | 252 (3.07%) | 2.69 (2.37 - 3.04) | 1.42 (1.23) |
| Oropharyngeal Pain | 237 (2.89%) | 1.02 (0.9 - 1.16) | 0.03 (-0.16) |
| Asthma | 231 (2.81%) | 0.87 (0.77 - 0.99) | -0.2 (-0.39) |
| Epistaxis | 191 (2.33%) | 1.04 (0.91 - 1.2) | 0.06 (-0.15) |
| Wheezing | 186 (2.27%) | 1.3 (1.13 - 1.5) | 0.38 (0.17) |
| Respiratory Failure | 170 (2.07%) | 1.09 (0.94 - 1.27) | 0.13 (-0.09) |
| Productive Cough | 168 (2.05%) | 1.39 (1.19 - 1.62) | 0.47 (0.25) |
| Pulmonary Oedema | 163 (1.99%) | 1.66 (1.42 - 1.94) | 0.73 (0.5) |
| Chronic Obstructive Pulmonary Disease | 154 (1.88%) | 1.3 (1.11 - 1.52) | 0.37 (0.14) |
| Lung Disorder | 146 (1.78%) | 1.26 (1.07 - 1.49) | 0.34 (0.1) |
| Nasal Congestion | 136 (1.66%) | 0.93 (0.79 - 1.1) | -0.1 (-0.35) |
| Rhinorrhoea | 136 (1.66%) | 0.83 (0.7 - 0.98) | -0.27 (-0.52) |
| Pleural Effusion | 135 (1.64%) | 1 (0.85 - 1.19) | 0 (-0.24) |
| Obstructive Airways Disorder | 129 (1.57%) | 4.52 (3.8 - 5.38) | 2.16 (1.9) |
| Rales | 126 (1.53%) | 10.01 (8.38 - 11.95) | 3.28 (3.02) |
| Pulmonary Embolism | 110 (1.34%) | 0.58 (0.48 - 0.7) | -0.78 (-1.06) |
| Pulmonary Fibrosis | 106 (1.29%) | 2.53 (2.09 - 3.06) | 1.33 (1.05) |
| Respiratory Disorder | 94 (1.14%) | 1.39 (1.13 - 1.7) | 0.47 (0.17) |
| Dysphonia | 84 (1.02%) | 0.59 (0.48 - 0.73) | -0.76 (-1.07) |
| Hypoxia | 83 (1.01%) | 1.05 (0.85 - 1.31) | 0.08 (-0.24) |
| Choking | 82 (1.00%) | 1.77 (1.42 - 2.2) | 0.82 (0.5) |
| Acute Respiratory Failure | 74 (0.90%) | 1.61 (1.28 - 2.02) | 0.68 (0.35) |
| Bronchiectasis | 73 (0.89%) | 4.54 (3.6 - 5.72) | 2.16 (1.83) |
| Dyspnoea At Rest | 59 (0.72%) | 7.63 (5.89 - 9.88) | 2.9 (2.52) |
| Respiratory Distress | 54 (0.66%) | 0.93 (0.71 - 1.22) | -0.1 (-0.49) |
| Pulmonary Hypertension | 54 (0.66%) | 1.19 (0.91 - 1.55) | 0.24 (-0.15) |
| Haemoptysis | 52 (0.63%) | 0.8 (0.61 - 1.05) | -0.32 (-0.72) |
| Throat Irritation | 48 (0.58%) | 0.46 (0.34 - 0.6) | -1.13 (-1.54) |
| Sputum Discoloured | 44 (0.54%) | 1.76 (1.31 - 2.36) | 0.81 (0.38) |
| Respiratory Arrest | 42 (0.51%) | 0.94 (0.69 - 1.27) | -0.09 (-0.54) |
| Sinus Disorder | 40 (0.49%) | 0.8 (0.59 - 1.1) | -0.31 (-0.76) |
| Pharyngeal Swelling | 40 (0.49%) | 1.5 (1.1 - 2.05) | 0.58 (0.13) |
| Pulmonary Congestion | 39 (0.47%) | 1.36 (0.99 - 1.86) | 0.44 (-0.02) |
| Acute Respiratory Distress Syndrome | 37 (0.45%) | 1.01 (0.73 - 1.39) | 0.01 (-0.46) |
| Throat Tightness | 36 (0.44%) | 0.61 (0.44 - 0.85) | -0.71 (-1.18) |
| Asphyxia | 35 (0.43%) | 1.71 (1.23 - 2.39) | 0.77 (0.29) |
| Throat Clearing | 35 (0.43%) | 3.24 (2.32 - 4.52) | 1.68 (1.2) |
| Respiration Abnormal | 35 (0.43%) | 1.96 (1.41 - 2.74) | 0.97 (0.49) |
| Tachypnoea | 34 (0.41%) | 1.13 (0.8 - 1.58) | 0.17 (-0.32) |
| Nasal Polyps | 34 (0.41%) | 3.54 (2.52 - 4.96) | 1.81 (1.32) |
| Emphysema | 33 (0.40%) | 1.57 (1.12 - 2.21) | 0.65 (0.15) |
| Pneumonitis | 31 (0.38%) | 0.47 (0.33 - 0.66) | -1.09 (-1.61) |
| Sneezing | 30 (0.37%) | 0.55 (0.38 - 0.78) | -0.87 (-1.39) |
| Sinus Pain | 30 (0.37%) | 4.51 (3.15 - 6.47) | 2.16 (1.63) |
| Pharyngeal Oedema | 29 (0.35%) | 0.98 (0.68 - 1.41) | -0.03 (-0.56) |
| Pulmonary Mass | 29 (0.35%) | 0.72 (0.5 - 1.04) | -0.47 (-1) |
| Paranasal Sinus Discomfort | 29 (0.35%) | 2.66 (1.85 - 3.83) | 1.4 (0.87) |
| Orthopnoea | 28 (0.34%) | 4.27 (2.94 - 6.2) | 2.08 (1.54) |
| Laryngeal Oedema | 26 (0.32%) | 2.06 (1.4 - 3.03) | 1.04 (0.48) |
| Organising Pneumonia | 26 (0.32%) | 2.13 (1.45 - 3.13) | 1.08 (0.53) |
| Pulmonary Vascular Disorder | 26 (0.32%) | 27.39 (18.32 - 40.94) | 4.65 (4.07) |
| Sleep Apnoea Syndrome | 24 (0.29%) | 0.51 (0.34 - 0.76) | -0.97 (-1.54) |
| Sinus Congestion | 23 (0.28%) | 0.74 (0.49 - 1.12) | -0.43 (-1.02) |
| Atelectasis | 22 (0.27%) | 1.25 (0.82 - 1.89) | 0.32 (-0.29) |
| Pneumothorax | 22 (0.27%) | 0.58 (0.38 - 0.89) | -0.78 (-1.38) |
| Pulmonary Alveolar Haemorrhage | 21 (0.26%) | 1.6 (1.04 - 2.45) | 0.67 (0.05) |
| Dry Throat | 19 (0.23%) | 0.8 (0.51 - 1.25) | -0.33 (-0.97) |
| Nasal Obstruction | 19 (0.23%) | 4.25 (2.7 - 6.68) | 2.07 (1.42) |
| Prolonged Expiration | 18 (0.22%) | 27.44 (16.92 - 44.5) | 4.65 (3.96) |
| Lung Hyperinflation | 17 (0.21%) | 8.23 (5.08 - 13.33) | 3.01 (2.32) |
| Bronchial Irritation | 16 (0.19%) | 43.31 (25.61 - 73.24) | 5.24 (4.49) |
| Choking Sensation | 15 (0.18%) | 1.21 (0.73 - 2.01) | 0.27 (-0.45) |
| Alveolitis | 15 (0.18%) | 6.13 (3.67 - 10.22) | 2.59 (1.86) |
| Nasal Dryness | 15 (0.18%) | 0.98 (0.59 - 1.62) | -0.04 (-0.76) |
| Respiratory Tract Congestion | 15 (0.18%) | 0.4 (0.24 - 0.66) | -1.33 (-2.05) |
| Nasal Discomfort | 14 (0.17%) | 0.77 (0.45 - 1.29) | -0.38 (-1.13) |
| Rhinitis Allergic | 14 (0.17%) | 1.44 (0.85 - 2.43) | 0.52 (-0.23) |
| Pleurisy | 14 (0.17%) | 1.13 (0.67 - 1.91) | 0.17 (-0.57) |
| Idiopathic Pulmonary Fibrosis | 14 (0.17%) | 1.34 (0.79 - 2.26) | 0.42 (-0.33) |
| Hypercapnia | 14 (0.17%) | 2.11 (1.25 - 3.58) | 1.07 (0.33) |
| Bronchitis Chronic | 12 (0.15%) | 2.25 (1.28 - 3.97) | 1.16 (0.36) |
| Asthmatic Crisis | 12 (0.15%) | 1.2 (0.68 - 2.12) | 0.27 (-0.54) |
| Laryngospasm | 11 (0.13%) | 1.99 (1.1 - 3.61) | 0.99 (0.15) |
| Pulmonary Arterial Hypertension | 11 (0.13%) | 0.28 (0.15 - 0.5) | -1.85 (-2.68) |
| Aspiration | 11 (0.13%) | 0.49 (0.27 - 0.89) | -1.02 (-1.85) |
| Yawning | 11 (0.13%) | 1.75 (0.97 - 3.16) | 0.8 (-0.03) |
| Respiratory Tract Oedema | 10 (0.12%) | 5.51 (2.95 - 10.29) | 2.44 (1.56) |
| Oropharyngeal Discomfort | 10 (0.12%) | 0.44 (0.23 - 0.81) | -1.19 (-2.07) |

Supplement Table 2. Signal strength of reports of rosuvastatin at the preferred term (PT) level in FAERS database.

| Preferred terms (PTs) | Case Numbers | ROR (95%Cl) | IC (IC_025_) |
| --- | --- | --- | --- |
| Dyspnoea | 1231 (16.49%) | 1.61 (1.52 - 1.7) | 0.68 (0.59) |
| Asthma | 768 (10.29%) | 5.11 (4.76 - 5.49) | 2.33 (2.23) |
| Cough | 648 (8.68%) | 1.62 (1.5 - 1.75) | 0.69 (0.58) |
| Wheezing | 460 (6.16%) | 5.65 (5.15 - 6.19) | 2.48 (2.34) |
| Dyspnoea Exertional | 225 (3.01%) | 4.16 (3.65 - 4.75) | 2.01 (1.85) |
| Bronchiectasis | 224 (3.00%) | 25.03 (21.89 - 28.63) | 4.57 (4.38) |
| Obstructive Airways Disorder | 213 (2.85%) | 13.1 (11.43 - 15.01) | 3.67 (3.47) |
| Productive Cough | 203 (2.72%) | 2.92 (2.54 - 3.35) | 1.54 (1.34) |
| Chronic Obstructive Pulmonary Disease | 196 (2.63%) | 2.87 (2.5 - 3.31) | 1.51 (1.31) |
| Rales | 187 (2.51%) | 26.24 (22.65 - 30.41) | 4.64 (4.42) |
| Interstitial Lung Disease | 123 (1.65%) | 1.92 (1.61 - 2.29) | 0.94 (0.68) |
| Pleural Effusion | 120 (1.61%) | 1.55 (1.29 - 1.85) | 0.63 (0.36) |
| Oropharyngeal Pain | 113 (1.51%) | 0.84 (0.7 - 1.01) | -0.25 (-0.52) |
| Pulmonary Embolism | 113 (1.51%) | 1.03 (0.86 - 1.24) | 0.05 (-0.22) |
| Haemoptysis | 95 (1.27%) | 2.55 (2.08 - 3.12) | 1.34 (1.05) |
| Nasal Congestion | 92 (1.23%) | 1.09 (0.89 - 1.34) | 0.13 (-0.17) |
| Lung Disorder | 90 (1.21%) | 1.35 (1.1 - 1.66) | 0.43 (0.13) |
| Sputum Discoloured | 90 (1.21%) | 6.29 (5.11 - 7.74) | 2.64 (2.33) |
| Dysphonia | 88 (1.18%) | 1.07 (0.87 - 1.32) | 0.1 (-0.2) |
| Rhinorrhoea | 79 (1.06%) | 0.83 (0.67 - 1.04) | -0.26 (-0.58) |
| Pulmonary Fibrosis | 78 (1.04%) | 3.22 (2.58 - 4.02) | 1.68 (1.35) |
| Hypopnoea | 76 (1.02%) | 15.53 (12.36 - 19.52) | 3.92 (3.58) |
| Catarrh | 75 (1.00%) | 56.74 (44.68 - 72.04) | 5.67 (5.33) |
| Nasal Oedema | 72 (0.96%) | 35.9 (28.26 - 45.59) | 5.07 (4.72) |
| Hypoxia | 68 (0.91%) | 1.5 (1.18 - 1.9) | 0.58 (0.23) |
| Pulmonary Mass | 65 (0.87%) | 2.82 (2.21 - 3.6) | 1.49 (1.13) |
| Dyspnoea At Rest | 56 (0.75%) | 12.56 (9.63 - 16.37) | 3.62 (3.23) |
| Respiratory Failure | 56 (0.75%) | 0.62 (0.48 - 0.81) | -0.68 (-1.06) |
| Epistaxis | 52 (0.70%) | 0.49 (0.37 - 0.65) | -1.02 (-1.42) |
| Pulmonary Oedema | 49 (0.66%) | 0.86 (0.65 - 1.14) | -0.21 (-0.62) |
| Obstructive Sleep Apnoea Syndrome | 46 (0.62%) | 24.77 (18.42 - 33.3) | 4.56 (4.13) |
| Lung Opacity | 42 (0.56%) | 10.89 (8.02 - 14.78) | 3.42 (2.97) |
| Aspiration | 42 (0.56%) | 3.29 (2.43 - 4.46) | 1.71 (1.27) |
| Sleep Apnoea Syndrome | 41 (0.55%) | 1.52 (1.12 - 2.06) | 0.6 (0.15) |
| Pulmonary Hypertension | 41 (0.55%) | 1.56 (1.15 - 2.12) | 0.64 (0.19) |
| Respiratory Distress | 37 (0.50%) | 1.11 (0.8 - 1.53) | 0.15 (-0.32) |
| Choking | 35 (0.47%) | 1.31 (0.94 - 1.82) | 0.38 (-0.1) |
| Throat Tightness | 33 (0.44%) | 0.97 (0.69 - 1.37) | -0.04 (-0.53) |
| Bronchospasm | 30 (0.40%) | 1.75 (1.22 - 2.5) | 0.8 (0.28) |
| Oropharyngeal Discomfort | 30 (0.40%) | 2.28 (1.59 - 3.26) | 1.18 (0.66) |
| Orthopnoea | 27 (0.36%) | 7.14 (4.89 - 10.45) | 2.82 (2.27) |
| Pneumothorax | 26 (0.35%) | 1.2 (0.81 - 1.76) | 0.26 (-0.3) |
| Pulmonary Congestion | 25 (0.33%) | 1.51 (1.02 - 2.24) | 0.59 (0.03) |
| Pharyngeal Oedema | 25 (0.33%) | 1.46 (0.99 - 2.16) | 0.54 (-0.02) |
| Sneezing | 24 (0.32%) | 0.76 (0.51 - 1.13) | -0.4 (-0.98) |
| Pleuritic Pain | 24 (0.32%) | 8.71 (5.82 - 13.04) | 3.1 (2.52) |
| Tachypnoea | 23 (0.31%) | 1.32 (0.88 - 1.99) | 0.4 (-0.19) |
| Throat Irritation | 22 (0.29%) | 0.36 (0.24 - 0.55) | -1.46 (-2.07) |
| Pulmonary Toxicity | 21 (0.28%) | 2.18 (1.42 - 3.34) | 1.12 (0.5) |
| Acute Respiratory Failure | 20 (0.27%) | 0.75 (0.48 - 1.16) | -0.41 (-1.04) |
| Pharyngeal Swelling | 20 (0.27%) | 1.3 (0.84 - 2.01) | 0.38 (-0.25) |
| Nasal Discharge Discolouration | 19 (0.25%) | 8.75 (5.56 - 13.77) | 3.11 (2.46) |
| Respiratory Disorder | 19 (0.25%) | 0.48 (0.31 - 0.76) | -1.04 (-1.69) |
| Sinus Pain | 18 (0.24%) | 4.67 (2.94 - 7.43) | 2.21 (1.55) |
| Lung Infiltration | 18 (0.24%) | 2.19 (1.38 - 3.48) | 1.13 (0.46) |
| Nasal Polyps | 17 (0.23%) | 3.05 (1.89 - 4.92) | 1.6 (0.92) |
| Rhinitis Allergic | 17 (0.23%) | 3.03 (1.88 - 4.88) | 1.59 (0.91) |
| Increased Upper Airway Secretion | 17 (0.23%) | 4.38 (2.72 - 7.07) | 2.12 (1.44) |
| Hypoventilation | 16 (0.21%) | 4.93 (3.01 - 8.06) | 2.29 (1.59) |
| Chronic Respiratory Disease | 15 (0.20%) | 31.03 (18.42 - 52.27) | 4.87 (4.13) |
| Sinus Congestion | 13 (0.17%) | 0.73 (0.42 - 1.25) | -0.46 (-1.23) |
| Organising Pneumonia | 13 (0.17%) | 1.84 (1.07 - 3.17) | 0.88 (0.1) |
| Reversible Airways Obstruction | 13 (0.17%) | 14.39 (8.29 - 24.98) | 3.81 (3.03) |
| Respiratory Tract Inflammation | 12 (0.16%) | 18.22 (10.24 - 32.42) | 4.14 (3.32) |
| Atelectasis | 12 (0.16%) | 1.18 (0.67 - 2.07) | 0.23 (-0.57) |
| Sinonasal Obstruction | 12 (0.16%) | 18.5 (10.4 - 32.92) | 4.16 (3.34) |
| Sputum Retention | 12 (0.16%) | 9.35 (5.28 - 16.56) | 3.2 (2.39) |
| Upper-Airway Cough Syndrome | 11 (0.15%) | 0.88 (0.49 - 1.59) | -0.18 (-1.02) |
| Respiratory Arrest | 10 (0.13%) | 0.39 (0.21 - 0.72) | -1.37 (-2.24) |
| Pneumonitis | 10 (0.13%) | 0.26 (0.14 - 0.49) | -1.93 (-2.8) |
